# Prognostic factors for renal function deterioration during palliative first-line chemotherapy for metastatic colorectal cancer: a retrospective study

**DOI:** 10.1007/s00520-022-07249-2

**Published:** 2022-07-02

**Authors:** Ah Reum Lim, Jwa Hoon Kim, Myung Han Hyun, Yeul Hong Kim, Soohyeon Lee

**Affiliations:** Division of Medical Oncology, Department of Internal Medicine, Korea University Anam Hospital, Korea University College of Medicine, 73, Goryeodae-ro, Seongbuk-gu, Seoul, 02841 Korea

**Keywords:** Metastatic colorectal cancer, Renal dysfunction, Palliative chemotherapy, Acute kidney injury

## Abstract

**Purpose:**

First-line choice of therapy is critical as it affects treatment decisions in later lines in patients with metastatic colorectal cancer (mCRC). We assessed changes in renal function for 1 year among patients diagnosed with mCRC who received first-line chemotherapy. We aimed to analyze the prognostic factors and effect of each chemotherapy regimen on the renal function of the patients.

**Methods:**

We retrospectively investigated patients with mCRC who were treated with a standard triplet regimen (FOLFOX/FOLFIRI with bevacizumab/cetuximab) in the first-line setting at Korea University Anam Hospital from 2015 to 2020. We checked renal function at 3-month intervals for 12 months. We calculated changes in eGFR (△eGFR, estimated glomerular filtration rate) and compared them with clinical factors such as age, sex, chronic disease, body mass index (BMI), disease status, baseline proteinuria, and first-line chemotherapy regimen.

**Results:**

Among 472 patients with mCRC, the median eGFR at baseline was 90.9 mL/min/1.73 m^2^; it was significantly lower (80.1 mL/min/1.73 m^2^, *p* < 0.001) at 12 months after chemotherapy initiation. Particularly, the eGFR of patients treated with FOLFIRI + bevacizumab was 74.9 mL/min/1.73 m^2^. The 1-year incidence rate of acute kidney injury (AKI) was 9.1%, with the lowest occurrence in patients receiving FOLFOX/cetuximab (2.1%) and the highest in those receiving FOLFIRI + bevacizumab (19.2%). Renal dysfunction was more frequent with FOLFIRI + bevacizumab as compared to the other regimens. Additionally, old age, low BMI, and proteinuria at baseline were also associated with a decreased eGFR.

**Conclusions:**

These findings can serve as important factors when selecting the first-line chemotherapy regimen for patients with mCRC.

**Supplementary Information:**

The online version contains supplementary material available at 10.1007/s00520-022-07249-2.

## Introduction

Metastatic colorectal cancer (mCRC) remains incurable in most cases and patients receive chemotherapy given in the non-curative setting to optimize symptom control and improve quality of life; however, large clinical trials that included patients healthy enough to receive chemotherapy demonstrated that intensive treatment with multiple systemic therapies may increase survival by 2 to 3 years [1–4]. First-line therapy consists of either oxaliplatin-based or irinotecan-based regimens, with or without additional targeted treatment. Comparison of FOLFOX and FOLFIRI as first-line treatments for mCRC demonstrated similar overall survival (OS). Most patients eventually received both regimens by transitioning from one regimen to other.

Although chemotherapy is effective in prolonging the overall survival of mCRC patients, it is necessary to optimize the drug selection and chemotherapy sequencing for patients’ long-term quality of life considering the potential adverse effects of chemotherapy. The drug nephrotoxicity is particularly important because the kidney is one of the most vulnerable organs, often leading to a poor prognosis and high in-hospital mortality. There are several causes of renal deterioration during cancer treatment. Regular computed tomography scans with contrast dye and the use of medications such as nephrotoxicity chemotherapy may deteriorate kidney function and contribute to increased morbidity and mortality in patients [5–7]. CRC is frequently diagnosed in the elderly—approximately 54% of the patients with mCRC are over 65 years of age [8]. In older patients, chemotherapy regimens, doses, and renal function have been identified as major risk factors for severe toxicity [9]. Acute kidney injuries (AKI) and their associated poor renal function may reduce the likelihood that mCRC patients receive optimal therapeutic management and supportive care.

Although several studies have reported that a substantial number of cancer patients with normal kidney function have significantly reduced creatinine clearance [10–13], few studies have focused on identifying risk factors for renal dysfunction during the long-term treatment of patients with mCRC. Hence, we assessed changes in renal function for 1 year in patients with mCRC and AKI incidence during therapy with standard triplet regimens (doublet cytotoxic chemotherapy plus a targeted agent). We also analyzed the impact of each chemotherapy regimen on renal function. The main aim of this retrospective study was to identify the risk factors, including the type of treatment regimen, for renal dysfunction in patients with mCRC during palliative first-line chemotherapy.

## Methods

### Study population

The study included patients with mCRC who received first-line, palliative chemotherapy and were diagnosed at the Division of Oncology of the Korea University Anam Hospital in Seoul, Korea, between January 1, 2015, and December 31, 2020. The analysis was limited to patients who had received standard triple therapy (doublet cytotoxic chemotherapy plus a target agent). Patients with the following characteristics were excluded: (i) treated with neoadjuvant or adjuvant chemotherapy, (ii) treated without target agents, (iii) administered a short duration of chemotherapy (< 3 cycles), (iv) had a history of first-line chemotherapy in another hospital, and (v) diagnosed with end-stage renal disease (ESRD) on dialysis. The study population was divided into four groups: (i) FOLFOX plus bevacizumab, (ii) FOLFOX plus cetuximab, (iii) FOLFIRI plus bevacizumab, and (iv) FOLFIRI plus cetuximab. We compared the renal function of the four treatment groups and monitored the changes over 1 year.

### Assessment of renal function

Renal function was assessed using two methods: change in estimated glomerular filtration rate (△eGFR) and incidence of AKI. The eGFR was calculated as follows for the male patients: eGFR (mL/min/1.73 m^2^) = 194 × Cr^−1.094^ × age^−0.287^. For the female patients, eGFR was calculated as follows (mL/min/1.73 m^2^) = 194 × Cr^−1.094^ × age^−0.287^ × 0.742. The serum creatinine (μmol/L) levels were obtained at five time points, including baseline and at 3-month intervals after chemotherapy initiation. △eGFR was calculated as [(eGFR at each time point)–(eGFR at baseline)/(eGFR at baseline) × 100].

AKI was defined as an increase of > 50% from the baseline serum creatinine (Cr) level per the Kidney Disease Improving Global Outcomes (KDIGO) criteria. We excluded the KDIGO criteria for serum Cr change within 7 days because many patients did not check serum Cr within 7 days before the onset of AKI. We compared the clinical characteristics between the AKI and non-AKI groups and identified the risk factors that could predict AKI development. We investigated the incidence of proteinuria during chemotherapy, which was defined as a sequential increase in protein level ≥ 1 + or exacerbation of pre-existing proteinuria, as determined using a dipstick urinalysis test.

### Data collection

Clinical and renal function data of the patients were collected retrospectively. Demographic factors, including age, sex, and body mass index (BMI), were collected. Tumor characteristics (location of the primary tumor, number of metastatic lesions, pathology of the tumor, molecular biomarkers (*KRAS*, *NRAS*, *BRAF* mutation, MSI (microsatellite instability) status), and treatment information (first-line chemotherapy regimens, duration of treatment, progression-free survival [PFS]) were obtained for all the patients. Information about comorbidities that might influence the development of nephrotoxicity (hypertension, diabetes, heart failure, liver disease, peripheral vascular diseases, cerebrovascular disease, chronic obstructive pulmonary disease (COPD), or asthma) was obtained. We also collected data about the consumption of potentially nephrotoxic medications before the occurrence of AKI, which included angiotensin-converting enzyme inhibitors (ACEi), angiotensin-receptor blockers (ARB), proton pump inhibitors (PPI), diuretics, nonsteroidal anti-inflammatory drugs (NSAIDs), and steroids. An ejection fraction of less than 50% on echocardiography was chosen as the criterion for diagnosing heart failure. Cerebrovascular disease was defined as a diagnosis of ischemic stroke or hemorrhage, and peripheral vascular disease was defined as a history of lower extremity deep vein thrombosis. Liver disease was defined as cirrhosis or alcoholic liver disease.

Data on the serum Cr (μmol/L) levels and proteinuria via urinalysis dipstick were collected at five time points at intervals of 3 months after chemotherapy initiation. The observation period for each patient was set as the time from the start of the first-line chemotherapy to 1 year.

### Statistical analysis

Descriptive statistics are presented as the median and interquartile ranges for numerical variables and numbers and percentages for categorical variables. Numerical variables between independent groups were analyzed using Student’s *t*-test in case of normal distribution and Mann–Whitney *U* test otherwise. Chi-square tests were used for between group comparisons. The comparison of the rates between the groups was performed using chi-square analysis. Logistic regression analysis was conducted to identify the determinant factors associated with the deterioration of renal function. Independent variables used were sex, age (< 65, ≥ 65 years), BMI (< 18.5, ≥ 18.5 kg/m^2^), presence of comorbidity, baseline eGFR (< 60, 60–90, ≥ 90 mL/min/1.73 m^2^), presence of proteinuria at baseline, and chemotherapy regimens. We initially conducted a univariate logistic regression analysis to identify the risk factors for AKI development. Statistically significant variables (*p* < 0.05) in the univariate analyses were included in the multivariate analysis. A backward stepwise model was used, with parameters at a *p*-value below 0.050. Data are presented as odds ratios (ORs) with 95% confidence intervals (CIs). An overall alpha error level of 5% was used to infer the statistical significance. All statistical analyses were conducted using SPSS v 20.0 (Statistical Package for the Social Sciences for Windows software, Chicago, IL, USA).

The study was conducted following the guidelines of the Declaration of Helsinki and approved by the Institutional Review Board of the Korea University Anam Hospital (IRB number: 2022AN0060). Informed consent was waived because of the retrospective nature of the study and there were minimal risks to subjects.

## Results

### Baseline characteristics

**Fig. 1:**
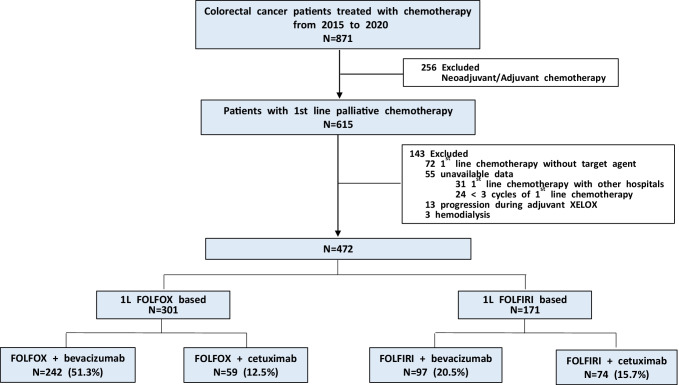
CONSORT diagram of the study process

This study included 472 patients with mCRC who received first-line chemotherapy (Fig. 1). The median follow-up period was 20.1 months (IQR, 12.5–33.2 months). Among them, 361 (66.4%) were men, 111 (33.6%) were women, and the median age was 62 years (IQR 54–71 years). Over half the patients had rectal cancer. The patients were not obese and had an average BMI of 23. Baseline eGFR was 95.4 mL/min/1.73 m^2^ (IQR, 82.2–106.6 mL/min/1.73 m^2^). Hypertension and diabetes mellitus were the frequent comorbidities (33.3% and 18.6%, respectively) (Table 1).

**Table 1:** Characteristics of patients

	FOLFOX + bev (*n* = 242)	FOLFOX + cet (*n* = 59)	FOLFIRI + bev (*n* = 97)	FOLFIRI + cet (*n* = 74)	Total (*n* = 472)
Age	62 (54–71)	63 (53–75)	61 (53–69)	61 (51–67)	62 (54–70)
Gender, *n* (%)					
Male Female	146 (60.3) 96 (39.7)	43 (72.9) 16 (27.1)	73 (75.3) 24 (24.7)	49 (66.2) 25 (33.8)	311 (65.9) 161 (34.1)
BMI	22.8 (20.7–25.3)	23.3 (21.7–25.3)	23.0 (21.4–25.1)	23.2 (20.9–25.6)	23.0 (21.0–25.3)
Hypertension, *n* (%)	102 (42.1)	22 (37.3)	30 (30.9)	24 (32.4)	178 (37.7)
Diabetes, *n* (%)	45 (18.6)	9 (15.3)	27 (27.8)	20 (27.0)	101 (21.4)
Primary location, *n* (%)					
Ascending colon	53 (21.9)	11 (18.7)	19 (19.6)	12 (16.2)	95 (20.1)
Descending colon	69 (28.5)	23 (39.0)	22 (22.7)	30 (40.5)	144 (30.5)
Rectum	120 (49.6)	25 (42.3)	56 (57.7)	32 (43.2)	233 (49.4)
Metastatic site, *n* (%)					
1	117 (48.3)	26 (44.1)	48 (49.5)	27 (36.5)	218 (46.2)
2	82 (33.9)	21 (35.6)	30 (309)	28 (37.8)	161 (34.1)
More than 2	43 (17.8)	12 (20.3)	19 (19.6)	19 (25.7)	93 (19.7)
Pathology, *n* (%)					
Adenocarcinoma Mucinous adenocarcinoma Others*	234 (96.7) 7 (2.9) 1 (0.4)	58 (98.3) 1 (1.7) 0 (0)	92 (94.8) 3 (3.1) 2 (2.1)	74 (100.0) 0 (0) 0 (0)	458 (97.0) 11 (2.3) 3 (0.6)
Molecular biomarker**, *n* (%)					
* KRAS* mutation * NRAS* mutation * BRAF* mutation MSI-H	132/221 (59.7) 7/209 (3.3) 10/178 (5.6) 2/132 (1.5)	0/57 (0.0) 0/57 (0.0) 4/50 (0.1) 2/31 ()	53/83 (63.9) 5/62 (8.1) 1/48 (2.1) 1/42 (2.4)	0/72 (0.0) 0/66 (0.0) 1/39 (2.4) 0/48 (0.0)	185/433 (42.7) 12/394 (3.0) 16/315 (5.1) 5/253 (2.0)
eGFR at baseline (mL/min/1.73m^2^)	91.0 (78.4–102.7)	84.5 (76.1–97.4)	90.1 (74.1–106.6)	94.7 (80.5–106.9)	90.9 (78.2–103.5)
Proteinuria at baseline, *n* (%) PFS	21 (8.7) 12.2 (7.6–20.2)	12 (20.3) 15.1 (8.2–23.2)	7 (7.2) 16.9 (9.2–30.4)	7 (9.5) 12.3 (7.1–19.3)	47 (10.0) 12.9 (7.9–22.7)

Data are frequency (percentage) or median (IQR, interquartile range)

*bev*, bevacizumab; *cet*, cetuximab; *BMI*, body mass index; *MSI-H*, microsatellite instability-high; *eGFR*, estimated glomerular filtration rate

^*^These include signet ring cell carcinoma, adenosquamous carcinoma, and squamous cell carcinoma

^**^Data not from all patients available

### Comparison of the eGFR changes

**Fig. 2:**
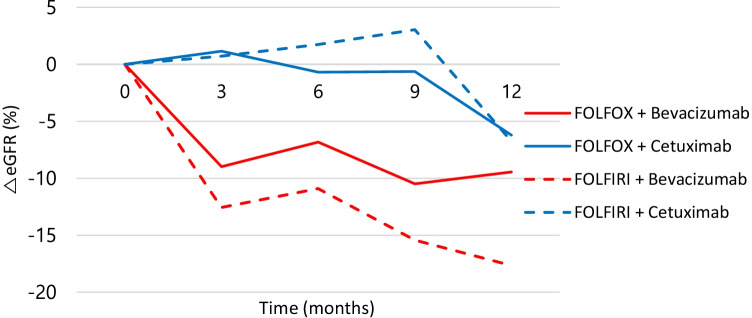
Changes in eGFR during first-line chemotherapy in patients with colorectal cancer. Data present changes as mean eGFR in patients with colorectal cancer up to 12 months after first-line chemotherapy. eGFR, estimated glomerular filtration rate

We compared the median eGFR change among the four chemotherapy regimens over 1 year. Overall, renal function decreased, regardless of the chemotherapy regimen. The decrease in eGFR was most significant in patients treated with FOLFIRI + bevacizumab compared to those receiving other regimens, ranging from 95.4 to 77.8 mL/min/1.73 m^2^ at 12 months (Fig. 2). We analyzed the proportion of eGFR < 30 or 60 mL/min/1.73 m^2^ between each chemotherapy group every 3 months. In the FOLFIRI + bevacizumab group, the proportion of patients whose eGFR decreased below 60 mL/min/1.73 m^2^ was 32.3% at 12 months, suggesting a strong correlation between chemotherapy regimen and impairment of renal function. The proportion of patients whose eGFR decreased below 30 mL/min/1.73 m^2^ was 1.9% at 12 months, and there was no difference between the groups (Fig. S1).

### Incidence of proteinuria and AKI during first-line chemotherapy

The 1-year incidence of proteinuria in patients with mCRC was 34.7%, with the highest proportion in patients treated with FOLFIRI + bevacizumab (47.4%), compared with the other three chemo-regimens. The overall incidence of proteinuria was higher in patients treated with bevacizumab than in those treated with cetuximab (Fig. S2).

**Fig. 3:**
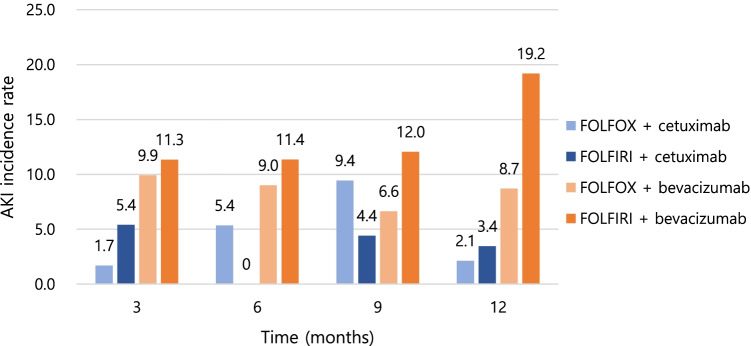
Incidence of AKI by 3 months during first-line chemotherapy in each chemotherapy group. AKI, acute kidney injury

The 1-year incidence of AKI in patients with mCRC was 9.1%, with the lowest value in patients treated with FOLFOX/cetuximab (2.1%) and the highest in those treated with FOLFIRI + bevacizumab (19.2%). The AKI incidence was the highest in patients treated with FOLFIRI + bevacizumab at any time point. Overall, the incidence of AKI increased in the following order: FOLFOX + cetuximab, FOLFIRI + cetuximab, FOLFOX + bevacizumab, and FOLFIRI + bevacizumab, except at 9 months (Fig. 3). Compared to the non-AKI group, patients with AKI were more likely to have a history of heart failure (7 [7.5%] vs. 11 [2.9%], *p* = 0.037) and proteinuria (15 [16.1%] vs. 32 [8.4%], *p* = 0.027).

Old age, low BMI, and FOLFIRI + bevacizumab regimen were more frequently associated with AKI (when compared with the non-AKI group) (AKI vs. no AKI in FOLFIRI + bevacizumab; 27 (29.0%) vs. 70 (18.5%), *p* = 0.024). Concomitant medications, comorbidities, and other chemotherapy regimens did not show any differences in AKI development (Table 2).

**Table 2:** Risk factors for acute kidney injury during 1st-line metastatic colorectal cancer treatment

	AKI (*n* = 93)	No AKI (*n* = 379)	*p*-value
Age	65 (58–71)	61 (53–70)	0.031
Gender, male, *n* (%)	63 (13.3)	248 (52.5)	0.674
BMI	22.4 (20.4–24.4)	23.3 (21.3–25.4)	0.013
eGFR at baseline (mL/min/1.73m^2^)	96.0 (84.5–115.6)	88.8 (77.2–102.2)	< 0.001
Proteinuria at baseline, *n* (%)*	15 (3.2)	32 (6.8)	0.027
Comorbidities, *n* (%)			
Cerebrovascular disease Heart failure COPD/asthma Hypertension Diabetes Liver disease Peripheral vascular disease	5 (1.1) 7 (1.5) 1 (0.2) 36 (7.6) 21 (4.4) 5 (1.1) 3 (0.6)	12 (2.5) 11 (2.3) 7 (1.5) 142 (30.1) 90 (19.1) 17 (3.6) 9 (1.9)	0.305 0.037 0.511 0.715 0.640 0.825 0.778
Concomitant medications, *n* (%)			
ACEi/ARB PPI Diuretics NSAIDs Steroid	34 (7.2) 28 (5.9) 5 (1.1) 9 (1.9) 0 (0)	89 (18.9) 96 (20.3) 15 (3.2) 20 (4.2) 10 (2.1)	0.951 0.348 0.543 0.113 0.222
1st chemotherapy regimen, *n* (%)			
FOLFOX + bevacizumab FOLFOX + cetuximab FOLFIRI + bevacizumab FOLFIRI + cetuximab	50 (10.6) 8 (1.7) 27 (5.7) 8 (1.7)	192 (40.7) 51 (10.8) 70 (14.8) 66 (14.0)	0.592 0.205 0.024 0.036

Data are frequency (percentage) or median (IQR, interquartile range)

*AKI*, acute kidney injury; *BMI*, body mass index; *eGFR*, estimated glomerular filtration rate; *COPD*, chronic obstructive pulmonary disease; *ACEi*, angiotensin-converting enzyme inhibitors; *ARB*, angiotensin receptor blocker; *PPI*, proton pump inhibitor; *NSAID*, non-steroidal anti-inflammatory drugs

### Prognostic factors for AKI

We performed a multivariate analysis using a logistic regression model to evaluate the independent risk factors for predicting post-chemotherapeutic AKI. In the univariate analysis, AKI was associated with age (≥ 65 vs. < 65 years; OR, 1.686; 95% CI, 1.068–2.662; *p* = 0.025), sex (male vs. female; OR, 1.229; 95% CI, 0.684–1.799; *p* < 0.001), BMI (≥ 18.5 vs. < 18.5 kg/m^2^; OR, 0.567; 95% CI, 0.354–0.909; *p* = 0.019), proteinuria at baseline (OR, 2.085; 95% CI, 1.077–4.037; *p* = 0.029), history of heart failure (OR, 2.723; 95% CI, 1.026–7.228; *p* = 0.044), and first-line chemotherapy regimen (FOLFIRI + bevacizumab vs. FOLFORI + cetuximab, OR, 3.182; 95% CI, 1.350–7.502; *p* = 0.008). The baseline eGFR, other comorbidities, concomitant medications, and other chemotherapy regimens were not associated with AKI. In the multivariable analysis, AKI was associated with age (≥ 65 vs. < 65, OR, 1.647; 95% CI, 1.028–2.638; *p* = 0.038), BMI (≥ 18.5 vs. < 18.5 kg/m^2^, OR, 0.559; 95% CI, 0.345–0.907; *p* = 0.018), baseline proteinuria (OR, 2.353; 95% CI, 1.159–4.777; *p* = 0.018), and first-line chemotherapy regimen (FOLFIRI + bevacizumab vs. FOLFORI + cetuximab; OR, 3.301; 95% CI, 1.377–7.910; *p* = 0.007) (Table 3).

**Table 3:** Univariate and multivariate analysis for acute kidney injury events

Factor	Univariate analysis			Multivariate analysis		
	OR	95% CI	*p*-value	OR	95% CI	*p*-value
Age (≥ 65 vs. < 65)	1.686	1.068–2.662	0.025	1.647	1.028–2.638	0.038
Gender (male vs. female)	1.229	0.684–1.799	< 0.001	1.061	0.638–1.765	0.820
BMI (≥ 18.5 vs. < 18.5)	0.567	0.354–0.909	0.019	0.559	0.345–0.907	0.018
Baseline eGFR (mL/min/1.73m^2^)						
< 60	1					
60– < 90	0.599	0.224–1.603	0.307			
≥ 90	1.435	0.563–3.662	0.450			
Baseline proteinuria	2.085	1.077–4.037	0.029	2.353	1.159–4.777	0.018
Comorbidities						
Cerebrovascular disease	1.738	0.597–5.060	0.311			
Heart failure	2.723	1.026–7.228	0.044	2.321	0.837–6.438	0.106
COPD/asthma	0.578	0.070–4.753	0.610			
Hypertension	1.054	0.661–1.680	0.825			
Diabetes	1.090	0.632–1.880	0.756			
Liver disease	1.210	0.435–3.369	0.715			
Peripheral vascular disease	1.370	0.364–5.165	0.642			
Concomitant medications						
ACEi/ARB	0.984	0.586–1.651	0.951			
PPI	1.270	0.770–2.093	0.349			
Diuretics	1.379	0.488–3.95	0.544			
NSAIDs	1.923	0.846–4.374	0.119			
1L chemotherapy regimen						
FOLFIRI + cetuximab	1			1		
FOLFOX + cetuximab	1.294	0.455–3.683	0.629	1.140	0.390–3.333	0.811
FOLFOX + bevacizumab	2.148	0.968–4.767	0.060	2.140	0.952–4.813	0.066
FOLFIRI + bevacizumab	3.182	1.350–7.502	0.008	3.301	1.377–7.910	0.007

*OR*, odds ratio; *CI*, confidence interval; *BMI*, body mass index; *eGFR*, estimated glomerular filtration rate; *COPD*, chronic obstuctive pulmonary disease; *ACEi*, angiotensin-converting enzyme inhibitors; *ARB*, angiotensin receptor blocker; *PPI*, proton pump inhibitor; *NSAID*, non-steroidal anti-inflammatory drugs

## Discussion

We investigated real-world data based on the effects of chemotherapy on renal dysfunction in patients with mCRC. Our findings demonstrated that the AKI incidence over 1 year was 9.1%. Among chemotherapy regimens, the FOLFIRI + bevacizumab showed the most decrease in eGFR and the highest AKI incidence. The significant prognostic factors for AKI were old age, BMI, proteinuria at baseline, and bevacizumab-containing regimens, especially FOLFIRI + bevacizumab.

In most cases of this study, renal function showed a tendency to decrease regardless of the chemotherapy regimen. The reasons for the renal function deterioration in mCRC patients are as follows: hypovolemia, post-renal obstruction with malignant infiltration, urinary tract obstruction, sepsis, and contrast agent nephropathy due to repetitive CT assessment and comorbidities due to the increase in the elderly population, as well as combined reasons. Renal deterioration of patients with metastatic cancer is a frequently occurring complication after chemotherapy. The IRMA (the Renal Insufficiency and Cancer Medications) study showed that kidney injury is common in people receiving anticancer therapy and that dose adjustment is required in these patients [14]. Since there is no significant difference in the therapeutic efficacy of chemotherapy regimens in mCRC, it is critical to preserve adequate organ function and avoid excessive toxicity when selecting the first chemotherapy regimen. There are few studies that predict the long-term toxicity by considering the patient’s basic characteristics and risk factors at the time of first chemotherapy regimen selection.

The anticancer drugs used among patients with mCRC are relatively less toxic to the kidneys. Oxaliplatin, a platinum complex anticancer drug, has lower renal toxicity than other platinum agents [15, 16]. Furthermore, irinotecan, a semi-synthetic derivative of camptothecin, has fewer renal complications because it is extensively subjected to hepatic metabolism and excreted into the bile [17, 18]. 5-FU, a pyrimidine antimetabolite, is also safe for renal function and does not require dose reduction due to renal dysfunction [19, 20]. There is still no worldwide consensus on renal dose adjustment for the use of 5-FU; however, some studies are aiming to standardize drug dose reduction in patients with renal dysfunction, including those on hemodialysis [19, 21]. Bevacizumab, a VEGF ligand inhibitor, can damage vascular endothelial cells. Its renal toxicity is mainly renovascular, including hypertension and proteinuria, which causes nephrotic syndrome and subsequently, decreased GFR [22]. Cetuximab, a monoclonal antibody targeting EGFR, is known to promote the progressive development of hypomagnesemia due to renal magnesium wasting [7, 23].

In our study, FOLFIRI + bevacizumab regimen was identified to worsen the most renal function. Irinotecan could induce diarrhea, with an incidence of 80–90%, among which grades 3 and 4 accounted for 39% [24], and intravascular volume depletion could induce pre-renal AKI. The combination of bevacizumab, which causes hypertension and proteinuria, could affect renal dysfunction more.

Some study has addressed the risk factors for AKI in gastrointestinal cancer. Li et al. [25] reported that diabetes, cancer type, anti-tumor treatment, alanine aminotransferase (ALT), serum creatinine, eGFR, serum uric acid, hypoalbuminemia, anemia, and abnormal sodium and potassium levels in patients with gastrointestinal cancers were risk factors for AKI; however, there were some differences from the risk factors in our study. It is thought that there were differences in terms of the study population, the frame of analysis, etc.

Our study has several limitations. It was a retrospective study performed at a single institute, and we did not follow the exact AKI definition of KDIGO with respect to the 7-day window period. The incidence of AKI in our study was 9.1%, which was lower than the previously reported incidences of AKI in patients with mCRC (9.2%, 20.3%, 22.5%, and 34.9%) [10, 26–28]. This may be due to the criteria used in our study pertaining to exclusion of patients who experienced disease progression during chemotherapy, the different definitions of AKI used, and the heterogeneity of the study population.

Nevertheless, we collected renal function serial eGFR data for 1 year after the initiation of chemotherapy and provided information about renal dysfunction as well as comorbidities and co-prescriptions to evaluate AKI risk factors in this population. Compared with previous studies that analyzed renal complications using a specific regimen [10, 28], this study analyzed the risk factors for renal dysfunction after the initiation of chemotherapy, focusing on the widely used 4 chemotherapy regimens of mCRC, i.e., FOLFIRI or FOLFOX combined with bevacizumab or cetuximab. We hope that it could be helpful for physicians to select 1st-line chemotherapeutic agents based on patients’ risk factors in terms of renal protection; in mCRC patients with high-risk factors, more careful monitoring is necessary when applying the first-line palliative chemotherapy to avoid or alleviate renal deterioration. These results provide useful information regarding toxicity and efficacy when selecting a first-line regimen for patients with mCRC.

## Supplementary Information

Below is the link to the electronic supplementary material.

Supplementary file1 (DOCX 48 KB)

## Data Availability

The data presented in this study are available on request from the author.
