# Supplementary material for: Prognostic factors for renal function deterioration during palliative first-line chemotherapy for metastatic colorectal cancer: a retrospective study
Source: Support Care Cancer. 2022 Jul 2;30(10):8129–37. doi: 10.1007/s00520-022-07249-2 (PMC9512747; doi:10.1007/s00520-022-07249-2)
Supplement: Supplementary file 1 — Supplementary file1 (DOCX 48 KB) [file 520_2022_7249_MOESM1_ESM.docx]

(a) FOLFOX + bevacizumab (b) FOLFOX + cetuximab

(c) FOLFIRI + bevacizumab (d) FOLFIRI + cetuximab

**Fig. S1** Distribution of eGFR in each chemotherapy group. Patients were classified according to their eGFR values (< 30, 30–59, 60–89, and > 90 mL/min/1.73m^2^). eGFR, estimated glomerular filtration rate.

**Fig. S2** Incidence of dipstick proteinuria during first-line chemotherapy in each chemotherapy group
